# Biometric Evaluation Using Body Measurements of Jiangyue Donkeys

**DOI:** 10.3390/ani16162584

**Published:** 2026-08-19

**Authors:** Wei Ren, Lei Zhao, Huaixing Yin, Qiong Wang, Lingling Liu

**Affiliations:** 1 College of Animal Science, Xinjiang Agricultural University, Urumqi 830052, China; rwei110@126.com (W.R.); 15276631506@163.com (L.Z.); yhxpwj@163.com (H.Y.); 2 Institute of Animal Husbandry, Xinjiang Academy of Animal Sciences, Urumqi 830011, China; wq_6973@163.com

**Keywords:** *Equus asinus*, morphometry, biometric traits, liveweight estimation, nonlinear models, animal growth

## Abstract

Body measurements provide a non-invasive basis for estimating live weight, yet their predictive value and age-related variation remain poorly characterized in female Jiangyue donkeys. Body weight (BW) and 11 morphometric traits were recorded in 484 females: withers height (WH), body length (BL), chest circumference (CC), cannon circumference (CAC), head length (HL), neck length (NL), chest width (CW), chest depth (CD), rump height (RH), rump length (RL), and rump width (RW). Descriptive statistics, correlation analyses, and regression analyses were performed using SPSS 27.0; stepwise regression was then applied to derive and validate BW prediction equations. Additionally, four nonlinear functions—Logistic, Gompertz, Brody, and von Bertalanffy—were fitted to cross-sectional BW data from 241 females sampled at different ages to characterize the population-level relationship between age and weight. BW was positively correlated with all morphometric traits (*p* < 0.01), with CC showing the strongest association. The optimal equations explained 91.8% and 84.4% of the variation in BW among growing and adult donkeys, respectively (both *p* < 0.01). Of the four nonlinear functions evaluated, the Brody model provided the closest fit (R^2^ = 0.99960) and yielded an estimated asymptotic mature weight of 191.049 kg. Because the age–weight analysis was based on cross-sectional observations, the resulting curve represents population-level variation rather than within-animal growth trajectories. Collectively, these models offer quantitative tools for live-weight estimation, growth assessment, and herd management in female Jiangyue donkeys.

## Simple Summary

China’s donkey industry is transitioning from its traditional reliance on draft animals toward diversified production systems centered on meat, hides, and milk. This shift has heightened the need for accurate assessment of body weight and growth, two parameters fundamental to precision feeding and genetic improvement. Yet, many farms lack access to weighing equipment or reliable field-based estimation methods, and the growth trajectories of several locally developed breeds remain poorly characterized. To address these limitations, female Jiangyue donkeys—a population developed in Xinjiang—were evaluated at multiple growth stages. Body weight and 11 morphometric traits were recorded, and their associations were quantified to derive parsimonious equations for predicting body weight from readily obtainable measurements. Four widely used nonlinear functions were then compared to determine which model most accurately captured the breed’s growth trajectory. The resulting equations enable rapid, equipment-free estimation of body weight under farm conditions; together with the optimized growth model, they establish an empirical basis for precision feeding, genetic-resource conservation, and selective breeding in Jiangyue donkeys.

## 1. Introduction

Donkeys have been raised in China for more than 4000 years, and the country recognizes 24 indigenous breeds. Donkey production remains a distinctive component of the national livestock sector [1,2,3]. However, mechanization and technological advances have progressively displaced donkeys from their traditional draught roles, contributing to a marked contraction of the national herd [4]. According to data released by the National Bureau of Statistics of China in 2025, the donkey population declined from 7.772 million in 2005 to 1.284 million in 2024—an 83.47% reduction over two decades [5]. Four indigenous breeds are currently classified as at risk of extinction, underscoring broader challenges in conserving donkey genetic resources while sustaining the domestic industry [6]. Concurrently, donkey production has shifted towards milk, hides, meat, and recreational riding, driven by growing consumer demand for donkey-derived products [7].

Since the 1970s, Guanzhong donkeys, a large-bodied breed indigenous to Shaanxi Province, have been introduced into Xinjiang and crossed with the smaller local Xinjiang donkey. This sustained crossbreeding programme gave rise to the Jiangyue donkey, a cultivated population characterized by environmental resilience, tolerance of low-quality roughage, and comparatively high production performance [8]. Jiangyue donkeys are now concentrated in Kashgar, Hotan, and neighbouring areas of Xinjiang. Because the population has not been formally recognized as a standardized breed, it is more appropriately classified as a cultivated commercial population [9]. Despite its value for livestock production and selective breeding in Xinjiang, the Jiangyue population remains poorly characterized quantitatively. In particular, associations between body weight and morphometric traits in females have received limited attention, and nonlinear models of population-level age–weight relationships have not been systematically compared. These knowledge gaps constrain evidence-based feeding, health management, and breeding decisions. Accurate live-weight estimates are required to determine drug dosages, formulate rations, adjust concentrate supplementation, and evaluate breeding performance. However, platform scales and other weighing equipment are unavailable on many farms. Consequently, body weight often cannot be measured reliably under field conditions, compromising both production efficiency and medication safety. Prediction equations based on readily obtainable body measurements could therefore provide an accessible alternative where direct weighing is impracticable. To characterize age-related variation in body weight, four established nonlinear growth functions—Logistic, Gompertz, Brody, and Von Bertalanffy—were evaluated. These models are widely used to describe livestock growth but differ in curve shape, growth-rate assumptions, and estimates of mature weight. Their comparative evaluation therefore enables selection of the function that most closely represents the early age–weight pattern of female Jiangyue donkeys.

Accordingly, three testable hypotheses were formulated: (i) body weight is positively associated with the principal morphometric traits of female Jiangyue donkeys, with chest circumference exhibiting the strongest correlation; (ii) multivariable equations derived through stepwise regression predict body weight with high accuracy across growth stages; and (iii) among the Logistic, Gompertz, Brody, and von Bertalanffy functions, the Brody model provides the closest fit to the population-level age–weight relationship between 1 and 14 months of age. These hypotheses were evaluated by quantifying stage-specific associations between body weight and morphometric traits, developing and validating body-weight prediction equations, and comparing the fit of four nonlinear functions to age-related variation in body weight.

## 2. Materials and Methods

### 2.1. Animals

Study animals were selected by cluster sampling from the foundation female herd of a large commercial donkey farm. All animals had unique identification numbers and complete husbandry records.

Animals were eligible for inclusion if they (i) were clinically healthy, with no evidence of systemic disease; (ii) exhibited no musculoskeletal abnormalities, including lameness or joint swelling, and had no injuries or deformities affecting the anatomical landmarks used for body measurements; and (iii) were not pregnant, thereby minimizing pregnancy-related effects on body weight and morphometric traits.

Animals were excluded if they (i) had a chronic debilitating condition or a documented history of illness during the preceding 3 months; (ii) had implausible or extreme measurement values that could not be resolved by checking the original records or repeating the measurements; or (iii) lacked complete age records in months.

Data were provided by Xinjiang Jinhuyang Animal Husbandry Co., Ltd. (Kashgar, China). The body-weight prediction dataset comprised 484 female Jiangyue donkeys and was used for correlation and regression analyses of body weight and morphometric traits. This dataset included 296 growing females aged 7 months to 3 years and 188 adult females aged 4–13 years. The age–weight dataset comprised 241 females aged 1–14 months and was used to compare nonlinear models of age-related variation in body weight. Of these animals, 120 were aged 1–6 months and were not included in the prediction dataset, whereas the remaining 121, aged 7–14 months, were also included in the growing group used for correlation and regression analyses. Thus, the two datasets overlapped by 121 animals. All animals were maintained at the same farm under uniform environmental and housing conditions and were managed according to the farm’s standardized feeding and husbandry protocols.

### 2.2. Measurement Methods and Indicators

Before the morning feeding, body weight (BW) was measured using a platform scale (Jiangsu Changjiang Weighing Apparatus Co., Ltd., Taizhou, China) with a resolution of 0.5 kg. Morphometric traits were measured using a flexible measuring tape (Delixi Electric LTD, Shanghai, China) or a measuring stick (Beijing Beimuxin Trade Center, Beijing, China), each with a resolution of 0.1 cm. The traits recorded were withers height (WH), body length (BL), chest circumference (CC), cannon circumference (CAC), head length (HL), neck length (NL), chest width (CW), chest depth (CD), rump height (RH), rump length (RL), and rump width (RW). All measurements were obtained in accordance with the procedures specified in the Technical Specification for Donkey Production Performance Determination [10], and the anatomical landmarks and measurement procedures for each trait are presented in Table 1. To minimize inter-observer variability, all measurements were performed by the same trained observer. Data collection was conducted from 8 July to 19 August 2025.

Each animal’s date of birth was verified against the farm’s birth and ear-tag identification records. Because birth dates were recorded to the nearest day, age at measurement was calculated from the interval between the recorded birth date and the measurement date and expressed in months. Only female Jiangyue donkeys were included because females constitute the farm’s core production population, and their body-weight and morphometric development is particularly relevant to reproductive and lactational management and breeding decisions. Males were not included because the number available was insufficient and their age distribution was too unbalanced to support reliable sex-specific analyses.

### 2.3. Statistical Analysis

Before the formal statistical analyses, each trait was screened for potential outliers using the mean ± 4 standard deviations (SD) criterion. A threshold of 4 SD, rather than the commonly used 3 SD, was chosen because the growing group covered a broad age range (7 months to 3 years) and therefore exhibited substantial age-related variation in body weight and morphometric traits. The wider threshold reduced the risk of incorrectly flagging biologically plausible observations arising from differences among developmental stages. Observations outside this range were checked against the original records and were excluded only when their validity could not be confirmed. Before Pearson’s correlation analysis, the distributions of all traits were evaluated visually using histograms and numerically using skewness and excess kurtosis. The histograms indicated approximately symmetric, unimodal distributions, and the absolute skewness and excess kurtosis values were <2 for all traits, indicating no substantial departures from normality. Because the variables were continuous, the relationships of interest were linear, and no marked distributional violations were identified, Pearson’s correlation coefficients were used to quantify the linear associations between body weight and morphometric traits.

SPSS Statistics 27.0 was used to perform descriptive statistics and Pearson correlation analyses for 484 female Jiangyue donkeys at different growth stages. Stepwise multiple linear regression was then used to identify candidate prediction equations relating BW to morphometric traits within the relevant growth-stage groups. The relationships among morphometric traits and their associations with BW were examined to characterize proportional development and body-conformation coordination. Strong, statistically significant positive correlations among major morphometric traits, together with strong associations with BW, were interpreted as evidence of coordinated development; however, these correlations were not considered direct measures of overall conformation quality. Because BW is relevant to feeding management, medication dosage, growth assessment, and breeding selection, equations for estimating BW were developed using morphometric traits that can be measured readily under farm conditions when weighing equipment is unavailable. Morphometric traits were entered as candidate independent variables, and BW was the dependent variable. The resulting regression equations were intended to provide practical estimates of BW from field-measurable body measurements.

OriginPro 2024 was used to fit four nonlinear growth models, namely the Logistic, Gompertz, Brody, and Von Bertalanffy models, to the age and BW data of 241 female Jiangyue donkeys aged 1–14 months. The model equations are presented in Table 2. For each model, the fitted population-level curve was plotted together with the observed age–BW data. These models were selected because they are widely used to describe animal growth and were specified according to previous studies [11,12,13,14]. The dataset was cross-sectional: each donkey was measured once, and different individuals represented the monthly ages from 1 to 14 months. Consequently, the fitted curves describe the population-level relationship between age and BW during the observed age range and should not be interpreted as longitudinal growth trajectories of individual animals.

## 3. Results

### 3.1. Descriptive Statistical Analysis of Body Weight and Body Measurements

Descriptive statistics were calculated for 296 growing female Jiangyue donkeys (hereafter, growing donkeys) and 188 adult female Jiangyue donkeys (hereafter, adult donkeys). Phenotypic data for the growing and adult groups are presented in Table 3 and Table 4, respectively. The groups differed in both sample size (296 versus 188) and developmental stage. Adult donkeys had higher mean body weight (BW) and body measurements than growing donkeys. The coefficient of variation (CV) quantifies variability relative to the mean: lower values indicate greater within-group uniformity, whereas higher values denote greater relative dispersion among individuals. In both developmental groups, BW had the highest CV of all traits, indicating that it exhibited the greatest relative phenotypic variability. By contrast, most morphometric traits had comparatively low CVs, reflecting limited within-group variation in body conformation. Taken together, these results support the use of BW as a reference trait in breeding programs.

### 3.2. Correlation Analysis of Body Weight and Body Measurements

**Figure 1:**
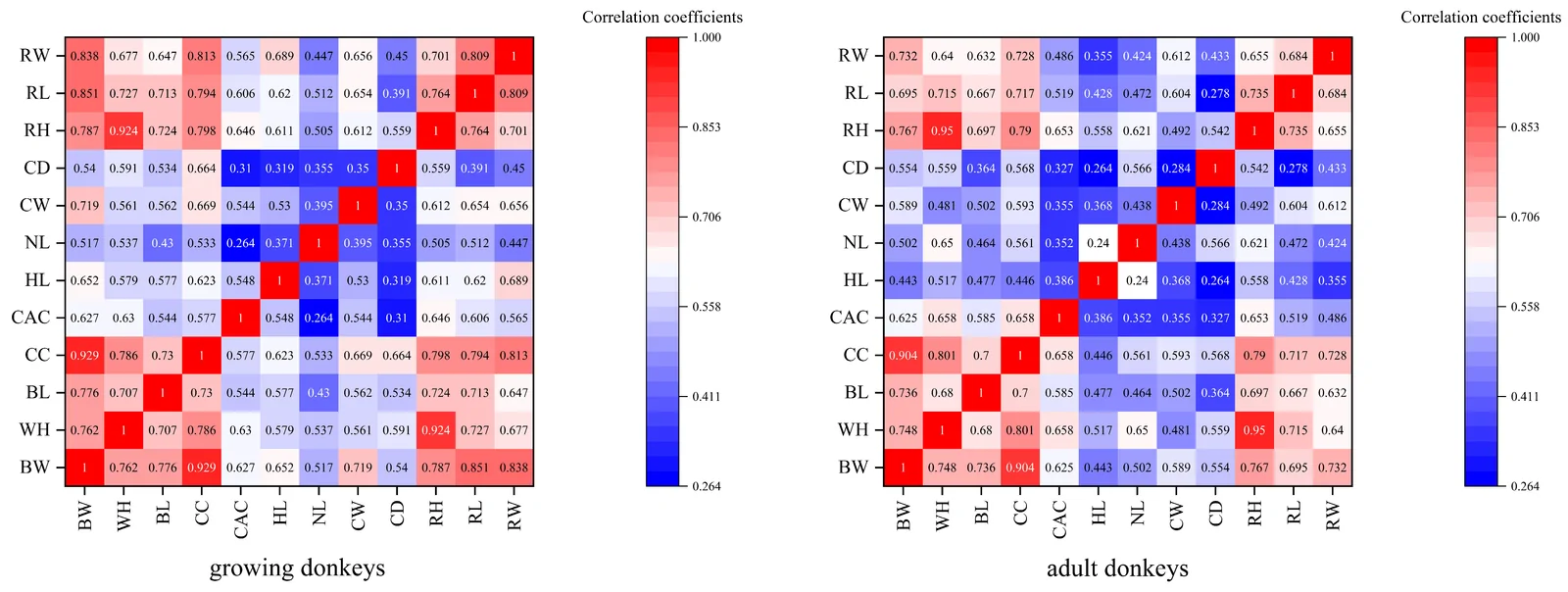
Heatmap of Pearson correlation coefficients among body weight and 11 body measurement traits in female Jiangyue donkeys. Color intensity indicates the strength of Pearson correlation coefficients. Darker red colors represent stronger positive correlations.

Correlation analyses of body weight (BW) and 11 morphometric traits were conducted to characterize their relationships across developmental stages and assess overall body conformation in female Jiangyue donkeys (Figure 1, Table 5 and Table 6). In both growing and adult donkeys, BW was positively correlated with every morphometric trait (*p* < 0.01); chest circumference showed the strongest association with BW, with correlation coefficients of 0.929 and 0.904, respectively. All pairwise correlations among the 11 morphometric traits were likewise positive and statistically significant (*p* < 0.01), indicating coordinated variation in body dimensions. The closest association was observed between withers height and rump height (0.924 in growing donkeys; 0.950 in adults), reflecting close correspondence between these two skeletal height measurements. By contrast, neck length and cannon circumference, as well as head length and neck length, were less strongly correlated, although both associations remained positive. Collectively, these patterns indicate coordinated growth of morphometric traits across both developmental stages, consistent with morphological uniformity and balanced body conformation in female Jiangyue donkeys.

### 3.3. Stepwise Regression Analysis of Body Weight and Body Measurements

Body weight (BW) was specified as the dependent variable, whereas 11 body measurements—withers height, body length (BL), chest circumference (CC), cannon circumference, head length, neck length, chest width (CW), chest depth (CD), rump height, rump length (RL), and rump width (RW)—were entered as candidate independent variables. Stepwise multiple linear regression was performed in SPSS 27.0 to identify the body measurements most strongly associated with BW and to derive prediction equations for growing and adult female Jiangyue donkeys. The results are presented in Table 7 and Table 8. Six variables were retained in the optimal model for growing donkeys: BL, CC, CW, CD, RL, and RW. The corresponding model was:

BW = 0.614BL + 2.501CC + 1.472CW − 0.863CD + 1.807RL + 0.953RW − 312.925 (1)

For adult donkeys, the optimal model included three variables—BL, CC, and RW:

BW = 0.774BL + 3.320CC + 1.461RW − 385.023(2)

The two models yielded coefficients of determination (R^2^) of 0.918 and 0.844 for growing and adult donkeys, respectively, indicating that the selected body measurements explained most of the variation in BW. Model-wide significance was subsequently assessed by analysis of variance (ANOVA), with the results presented in Table 9. Both regression models were statistically significant: F_1_ = 541.738 for growing donkeys and F_2_ = 331.459 for adult donkeys (*p* < 0.01 for both). These results confirmed significant linear relationships between BW and the selected combinations of body measurements. All models were derived exclusively using stepwise multiple linear regression; principal component analysis (PCA) was not performed.

### 3.4. Residual Analysis and Model Diagnosis

Stepwise multiple regression was used to derive the BW prediction equations. Despite its widespread application in animal science, this approach has recognized limitations, including susceptibility to overfitting, coefficient instability across samples, and inflated Type I error rates. Given the intended use of these equations as on-farm BW estimation tools, their statistical robustness and predictive reliability require rigorous evaluation.

**Figure 2:**
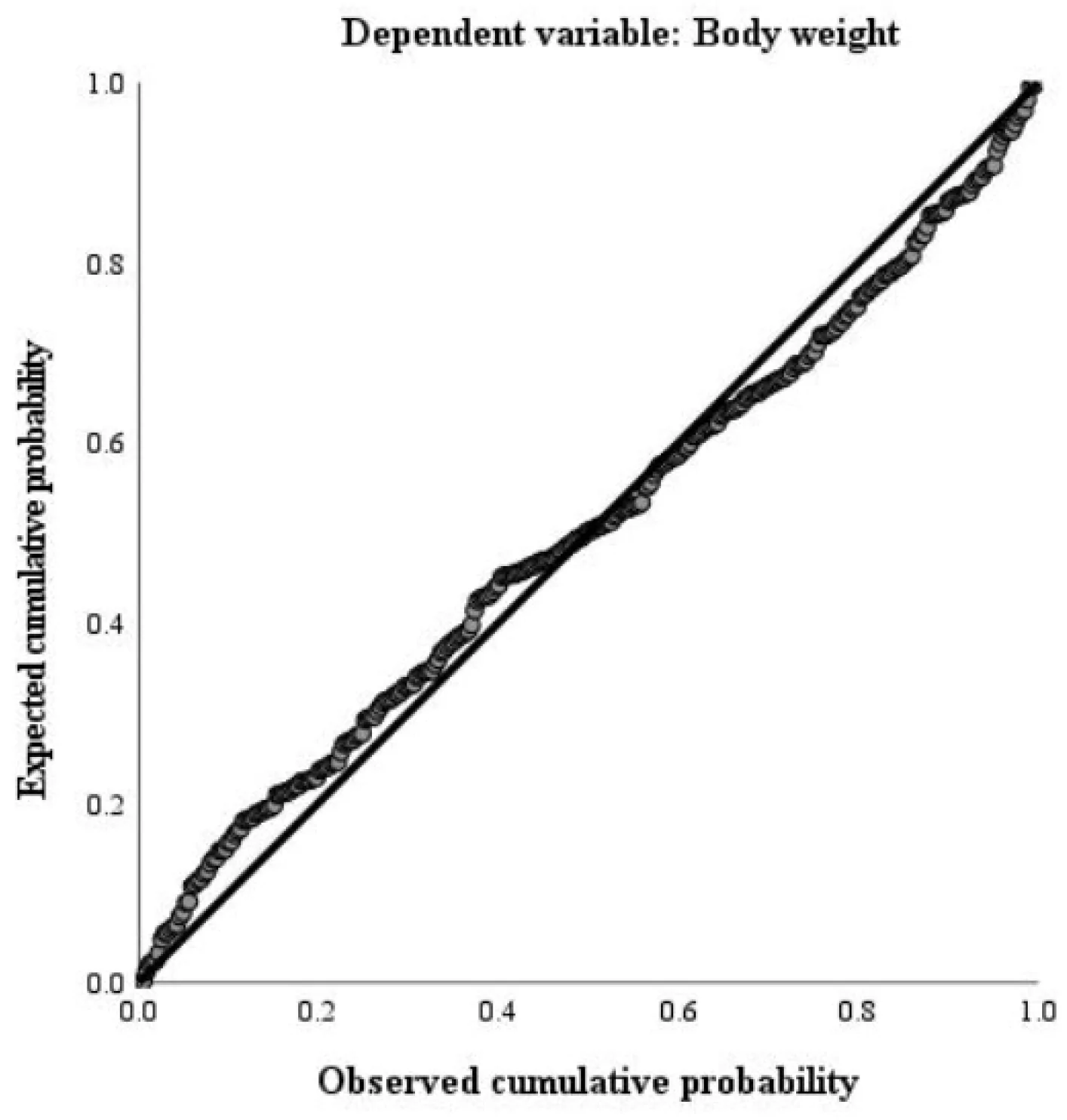
P-P plot of standardized residuals for body weight regression of growing female Jiangyue donkeys.

**Figure 3:**
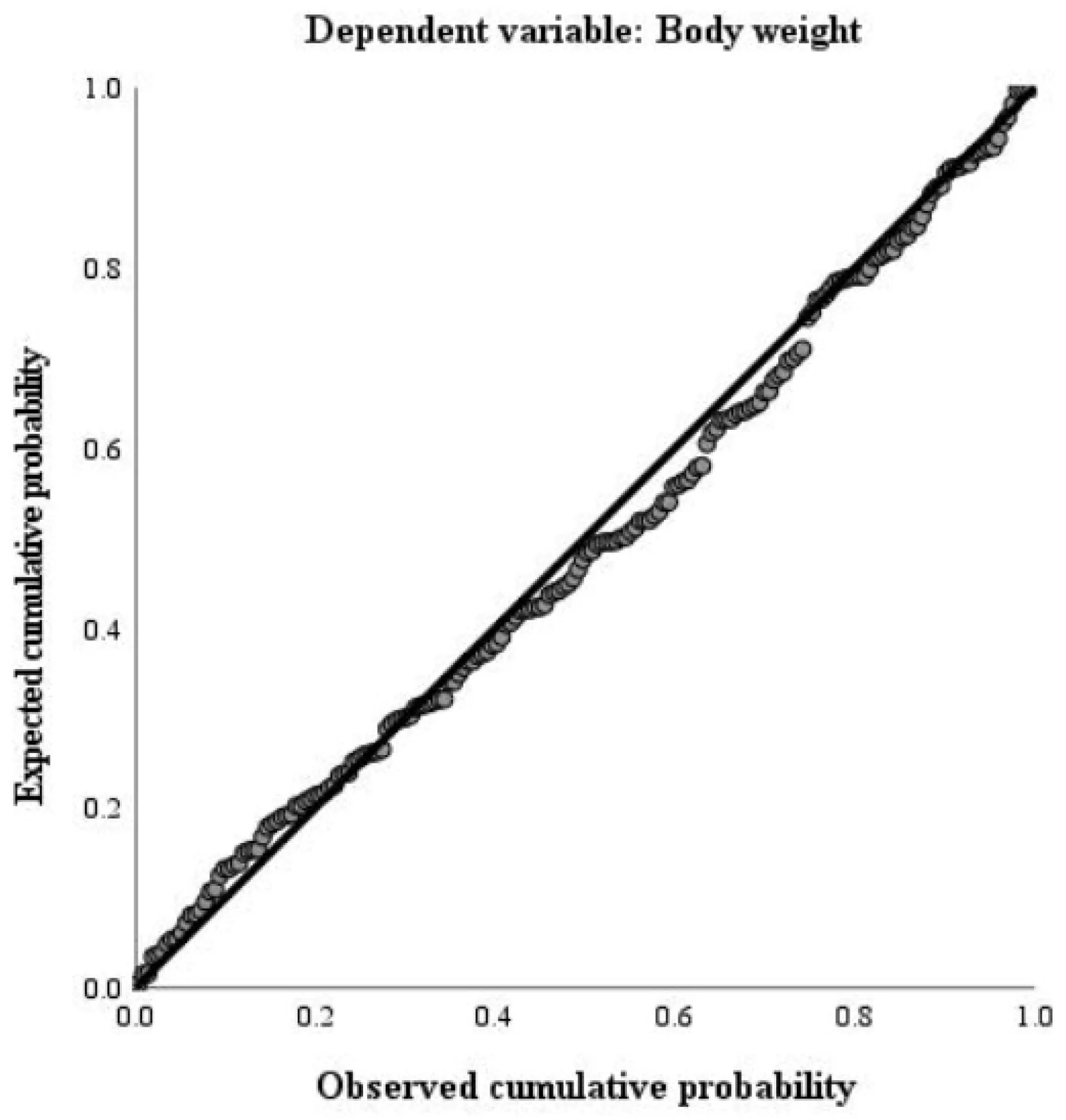
P-P plot of standardized residuals for body weight regression of adult female Jiangyue donkeys.

**Figure 4:**
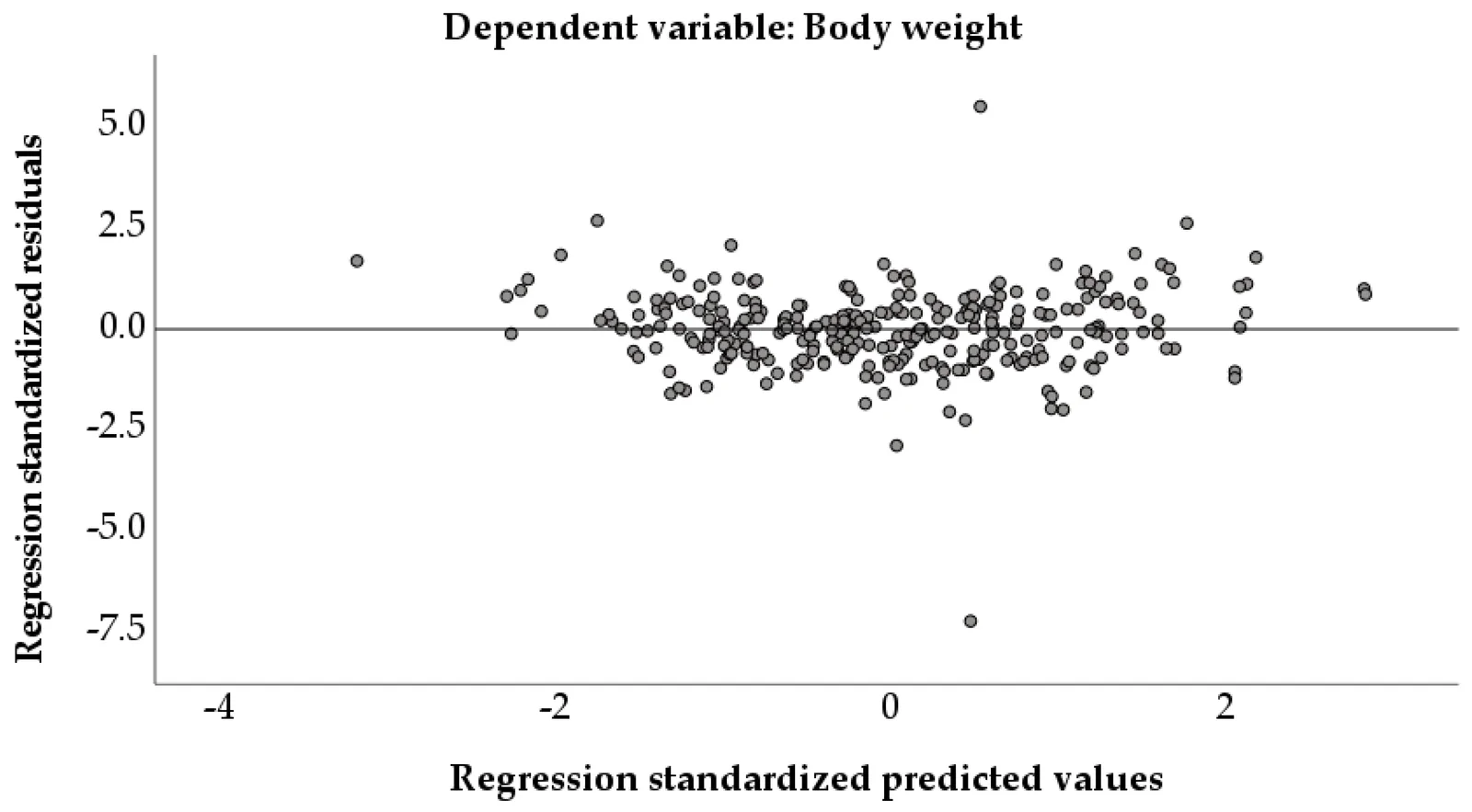
Scatter plot of standardized residuals for body weight regression of growing female Jiangyue donkeys.

**Figure 5:**
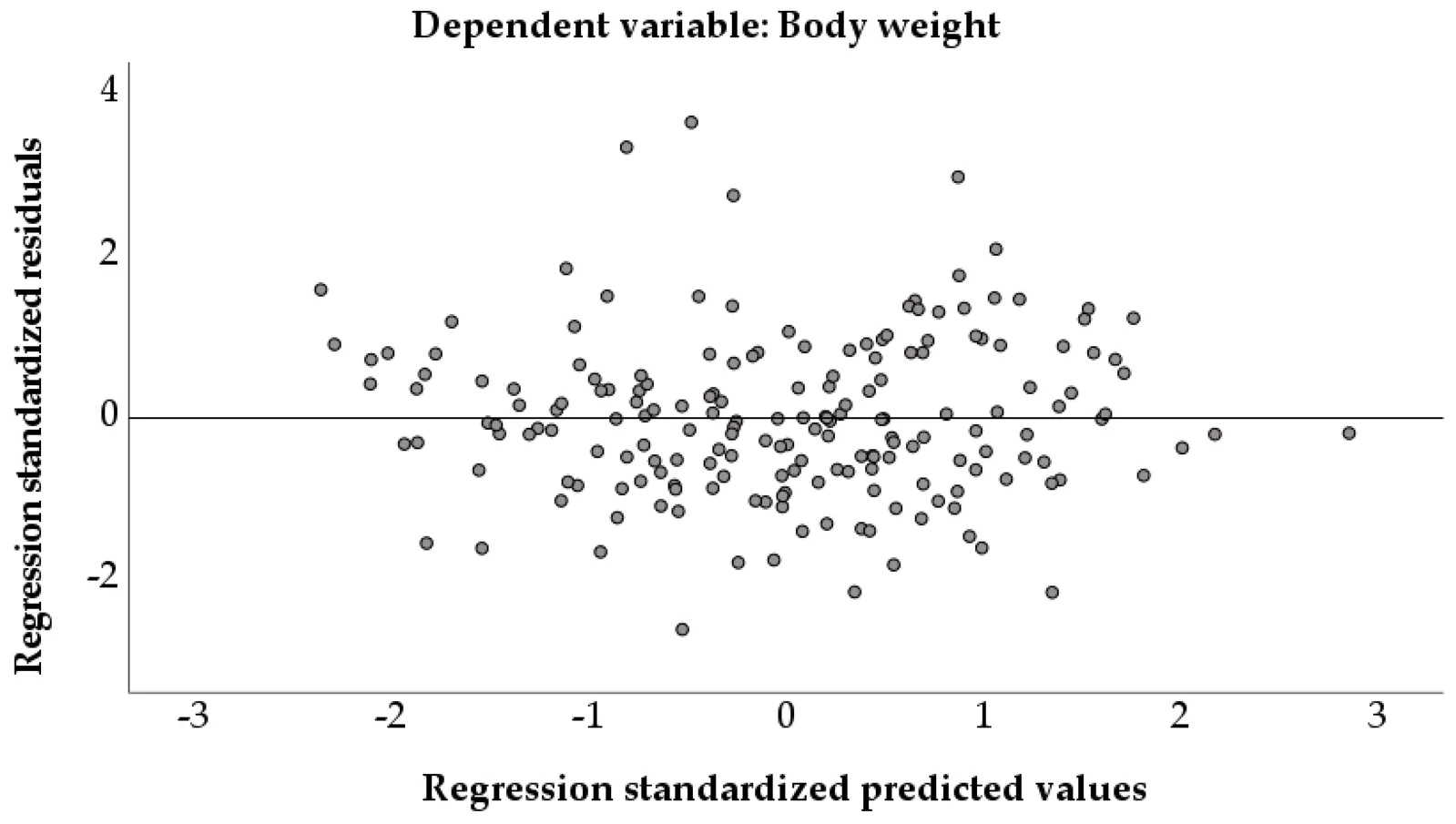
Scatter plot of standardized residuals for body weight regression of adult female Jiangyue donkeys.

Systematic residual analyses and multicollinearity diagnostics were conducted for both optimal regression models to assess the assumptions of multiple linear regression. In the P-P plots (Figure 2 and Figure 3), the standardized residuals closely followed the diagonal reference line, indicating approximate normality. The residual-versus-predicted-value plots (Figure 4 and Figure 5) showed that the standardized residuals were distributed approximately evenly around zero, with no evident systematic pattern, providing no visual evidence of substantial heteroscedasticity. Residual autocorrelation was assessed using the Durbin-Watson statistic, which ranges from 0 to 4. Values below 2 generally suggest positive autocorrelation, values above 2 suggest negative autocorrelation, and values near 2 indicate little or no first-order autocorrelation [15]. As shown in Table 7, the Durbin-Watson statistics were 1.878 and 1.619 for the growing and adult donkey models, respectively. These results provided no clear evidence of substantial residual autocorrelation, although the value for the adult model deviated further from 2. In addition, the variance inflation factors (VIFs) of all retained predictors were below 6, indicating that severe multicollinearity was not evident based on the adopted threshold.

Potentially influential observations were further evaluated using Cook’s distance and centered leverage values obtained from the regression analyses in SPSS 27.0. For the growing-donkey model, the maximum Cook’s distance and centered leverage value were 0.208 and 0.224, respectively; the corresponding values for the adult-donkey model were 0.062 and 0.094. A small number of observations exhibited relatively high leverage, potentially reflecting the broad age ranges within the growing (7 months to 3 years) and adult (4 to 13 years) groups and the resulting heterogeneity in predictor values. However, the maximum Cook’s distances for both models were well below the commonly used reference value of 1, suggesting that no individual observation exerted an unduly large influence on the estimated regression coefficients. Overall, these diagnostics provided no evidence of highly influential observations and supported the stability of the fitted models.

To further assess the stability of the coefficients obtained through stepwise regression, bootstrap resampling was performed for all predictors retained in the final models, using 1000 resamples. In this procedure, samples were repeatedly drawn with replacement from the original dataset to characterize the sampling variability of the estimated regression coefficients. The bootstrap 95% confidence intervals for all retained predictors excluded zero and were highly consistent with the corresponding original coefficient estimates. These findings indicate that the associations between the selected body measurements and BW were stable under internal resampling and provide supportive evidence for the robustness of the fitted models.

In summary, the diagnostic analyses provided no substantial evidence of violations of the principal assumptions of multiple linear regression, including non-normality, heteroscedasticity, residual autocorrelation, severe multicollinearity, or undue influence by individual observations. The bootstrap results also supported the stability of the coefficients under internal resampling. Collectively, these findings support the use of the established equations for estimating the body weight of Jiangyue donkeys and provide a statistical basis for their potential application in precision feeding management. However, external validation in independent herds and under practical farm conditions is still needed to confirm their generalizability and operational reliability.

### 3.5. Validation of Regression Models

To assess potential overfitting, a fixed random seed was specified in SPSS 27.0 before randomly partitioning each dataset into training and test sets at an 8:2 ratio. A seed of 123,456 was used for the growing-donkey dataset (*n* = 296), and a seed of 654,321 was used for the adult-donkey dataset (*n* = 188). For the growing-donkey dataset, 236 observations were assigned to the training set and 60 to the test set. Stepwise regression was then repeated using only the training data, and the selected predictors were identical to those identified in the full sample. The resulting equation was: BW = 0.581BL + 2.546CC + 1.480CW − 0.710CD + 1.876RL + 0.768RW − 317.939 (R^2^ = 0.915, *p* < 0.01), which was highly consistent with the equation obtained from the full sample (R^2^ = 0.918). When applied to the test set, the model yielded an R^2^ of 0.933, an RMSE of 8.32 kg, an MAE of 6.83 kg, and a MAPE of 4.28% (Table 10). For the adult-donkey dataset, 146 observations were assigned to the training set and 42 to the test set. The stepwise model fitted to the training data retained the same predictors as the full-sample model and produced the following equation: BW = 0.648BL + 3.428CC + 1.511RW−385.956 (R^2^ = 0.843, *p* < 0.01), compared with R^2^ = 0.844 for the full-sample model. Applied to the test set, the model produced an R^2^ of 0.847, an RMSE of 13.71 kg, an MAE of 11.88 kg, and a MAPE of 5.39%. The comparable training- and test-set performance, together with the consistency of the selected predictors and regression coefficients, provided no clear evidence of substantial overfitting in either model. The slightly higher R^2^ observed for the growing-donkey test set than for its training set may reflect random variation in the distribution of observations after partitioning, particularly given the relatively limited test-set size.

To obtain a more robust assessment of model generalizability and reduce dependence on a single random train–test partition, repeated 10-fold cross-validation was performed 10 times for both models in R, using fixed random seeds of 123,456 for the growing-donkey model and 654,321 for the adult-donkey model to ensure reproducibility. As shown in Table 11, the mean cross-validated R^2^ values were 0.9187 ± 0.0560 for the growing-donkey model and 0.8476 ± 0.0679 for the adult-donkey model. The corresponding mean RMSE values were 9.32 ± 2.87 kg and 14.44 ± 2.79 kg, respectively. The mean cross-validated R^2^ values were similar to those obtained from the full-sample models (R^2^ = 0.918 and 0.844, respectively), indicating generally consistent predictive performance across resampled subsets. Although the R^2^ values and RMSEs varied across repetitions, the variability in RMSE was moderate relative to the respective mean values, suggesting that model performance was not strongly dependent on a particular data partition. The slightly higher test-set R^2^ observed for the growing-donkey model in the single random split may therefore reflect sampling variation rather than a meaningful difference in predictive ability. Overall, the repeated cross-validation results provided supportive evidence that the model structures and predictive performance were reasonably stable under internal resampling, with no clear indication of substantial overfitting. Accordingly, the equations estimated from the full samples were retained as the final equations for field application.

### 3.6. Fitting of Age-Related Weight Patterns with Different Models

**Figure 6:**
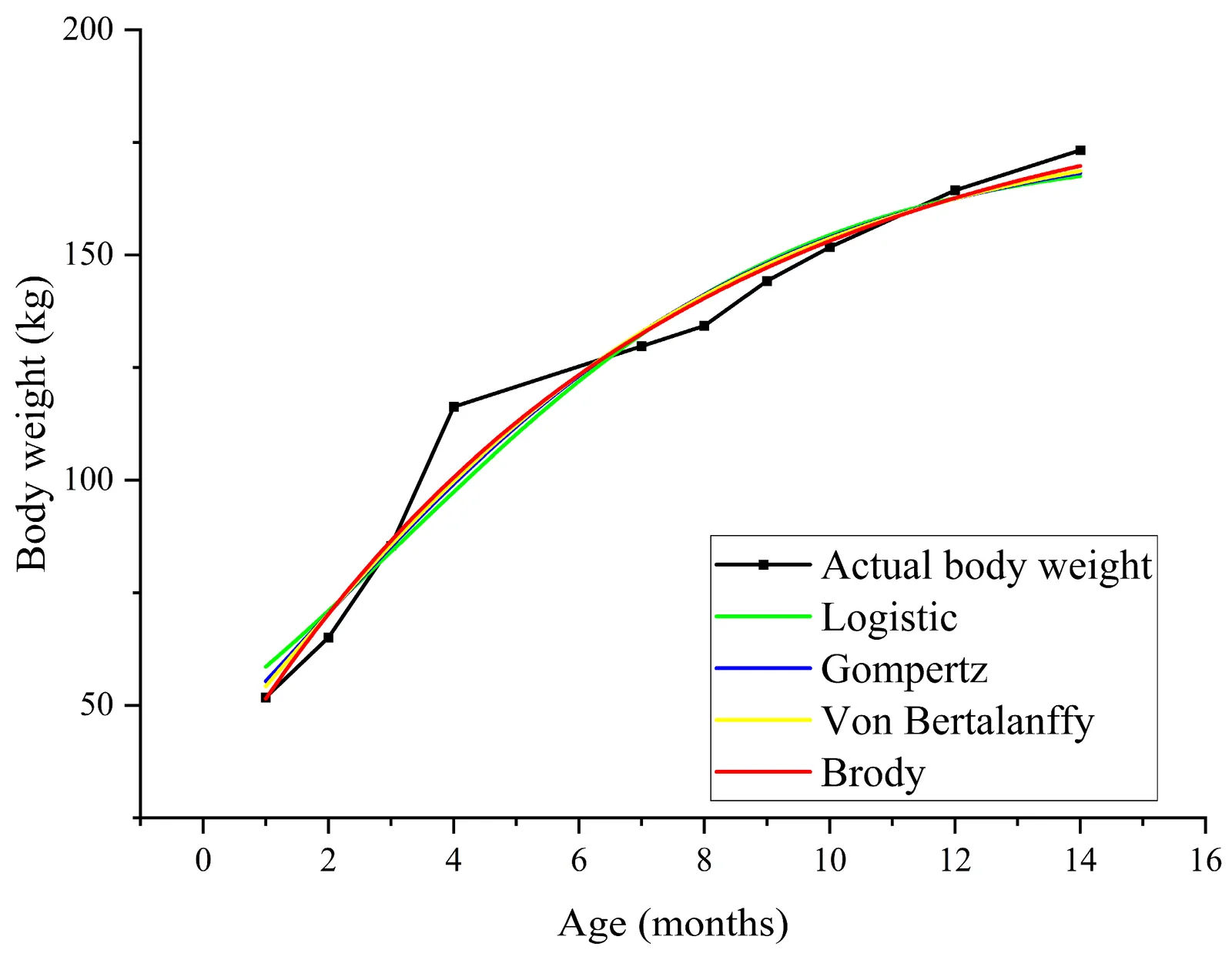
Cross-sectional age–weight relationship models for female Jiangyue donkeys.

In this study, body weight was recorded for female Jiangyue donkeys aged 1–14 months. Owing to insufficient valid sample sizes at some ages, only measurements obtained at 1, 2, 3, 4, 7, 8, 9, 10, 12, and 14 months were included in the growth-curve analysis. The mean body weights and standard deviations of the 241 female donkeys at the included ages are presented in Table 12. Four nonlinear growth models, namely the Logistic, Gompertz, Brody, and Von Bertalanffy models, were fitted to the mean body weights. As shown in Table 13 and Figure 6, mean body weight increased rapidly during the first four months of age, followed by a gradual reduction in the apparent growth rate. All four models described the observed age-related pattern reasonably well, with R^2^ values exceeding 0.96418 and statistically significant overall fits (*p* < 0.01). Based on a joint comparison of R^2^, RMSE, AIC, and BIC, the Brody model provided the most favorable overall fit. It produced the highest R^2^ (0.99960) and the lowest RMSE (5.868 kg), while its AIC (55.960) and BIC (58.517) were close to the lowest values obtained among the candidate models. The Brody model was therefore selected as the preferred model for describing the relationship between age and body weight in the female Jiangyue donkeys examined in this study. The asymptotic weight estimated by the Brody model was 191.049 kg. This estimate represents a model-derived theoretical mature weight rather than a directly observed adult weight and should be interpreted cautiously because the available data extended only to 14 months of age. Consequently, although the Brody model closely described body weight within the observed age range, its use for predictions beyond 14 months may involve considerable extrapolation uncertainty.

## 4. Discussion

### 4.1. Descriptive Statistical Analysis of Body Weight and Body Measurements

Body measurements and body weight provide direct phenotypic indices of growth, development, and production potential in donkeys [16]. Body weight exhibited the greatest variation among all traits measured in growing and adult female Jiangyue donkeys, with coefficients of variation (CVs) of 19.04% and 16.00%, respectively. Comparable patterns have been documented in Dezhou [17] and Xinjiang donkeys [18], indicating pronounced interindividual variation in donkey body weight. Its particularly broad phenotypic range may therefore support its use as a selection criterion in breeding programmes. Among the morphometric traits, rump width showed the highest CV in growing donkeys (9.22%). This variation may reflect heterogeneity in the development of hindquarter bones and musculature, with differences in individual growth trajectories becoming particularly evident in hindquarter width during this developmental stage. By contrast, chest width was the most variable morphometric trait in adult donkeys (CV = 9.16%). Such variation may reflect phenotypic heterogeneity within the population and, potentially, differences in feeding and management that affect thoracic development, thereby indicating scope for selective improvement. All remaining body measurements had CVs below 9%, reflecting comparatively limited phenotypic variation.

### 4.2. Correlation Analysis of Body Weight and Body Measurements

Correlation analysis is used to study the correlation between variables, test their independence and positive/negative correlation, and the correlation coefficient measures the strength of their association [19]. At both the growing and adult stages, chest circumference showed the strongest correlation with body weight in female Jiangyue donkeys. Similar findings have been reported for Xinjiang [18], Dezhou [20], West African [21], and Miranda donkeys [22]. The present results, however, differed to some extent from those reported for Jiangyue donkeys by Xiao Haixia et al. [23]. This discrepancy may reflect differences in sample size, sex and age composition, feeding conditions, or other population-specific factors. Withers height and rump height were the most strongly correlated morphometric traits at both developmental stages, indicating close coordination between these two measures of longitudinal skeletal growth. This relationship suggests a high degree of proportionality in the body conformation of female Jiangyue donkeys. By contrast, head length, neck length, and cannon circumference were less strongly correlated with body weight, suggesting that they capture less of the variation in overall body mass than other measurements. One possible explanation is that these traits primarily reflect localized skeletal dimensions rather than overall body size and muscular development.

### 4.3. Stepwise Regression Analysis of Body Weight and Body Measurements

Stepwise regression selects predictors according to predefined significance criteria, yielding a parsimonious model of the relationship between a dependent variable and its covariates [24]. Applied separately to growing and adult female Jiangyue donkeys, this procedure produced stage-specific equations for body-weight estimation. The model for growing donkeys retained six predictors—body length, chest circumference, chest width, chest depth, rump length, and rump width—and explained 91.8% of the variation in body weight (R^2^ = 0.918). By contrast, the adult model retained only body length, chest circumference, and rump width, accounting for 84.4% of the observed variation (R^2^ = 0.844). The inclusion of these three measurements at both developmental stages identifies them as consistent predictors of body weight and potentially informative phenotypic criteria for the selection and breeding of Jiangyue donkeys. Using stepwise regression, Xiao Guoliang et al. [18] identified chest circumference and body length as the principal predictors of body weight in adult female Xinjiang donkeys, partially corroborating the present findings. In northwestern Nigerian donkeys, however, John et al. [25] found that withers height and chest circumference provided the strongest predictors of body weight in adult females. Their model thus differed from the adult Jiangyue donkey model reported here. Similarly, Xiao Haixia et al. [26] retained withers height, body length, and chest circumference in age-specific equations developed for Jiangyue donkeys. The discrepancy may reflect differences in the sex composition of the sampled animals and the range of morphometric traits evaluated. Collectively, these studies demonstrate that body measurements can predict donkey body weight, although the predictors retained vary across populations, developmental stages, and study designs.

Chest depth exhibited a negative regression coefficient in growing donkeys (β = −0.863), despite its positive correlation with body weight (r = 0.540). This sign reversal is likely attributable to multicollinearity among the predictors: chest depth, chest circumference, and chest width capture distinct dimensions of thoracic volume and are therefore strongly intercorrelated. Rather than negating the underlying contribution of chest depth to body weight, the negative coefficient represents its partial effect after adjustment for body proportions and other correlated thoracic measurements. To determine whether this estimate arose from random sampling variation, the regression model was validated by bootstrap resampling with 1000 iterations. The 95% bootstrap confidence interval for the chest-depth coefficient ranged from −1.474 to −0.358, lying entirely below zero. The negative coefficient was therefore stable within the fitted model and dataset, reflecting the conditional association of chest depth with body weight after chest circumference, chest width, and the remaining predictors had been held constant. All variance inflation factors were below 6, indicating that multicollinearity did not compromise overall model stability, although the coefficients of individual thoracic measurements should be interpreted conditionally. Chest circumference, body length, and rump width capture complementary components of body mass—thoracic capacity, longitudinal skeletal development, and hindquarter muscularity, respectively. Accordingly, breeding decisions should integrate overall conformational harmony while prioritizing females with a large chest circumference, moderate body length, and well-developed rump width, thereby promoting weight-gain efficiency without compromising structural balance.

The regression models developed here are population-specific: both model development and internal validation were conducted within a single population under a common management system, and no independent external dataset was available for validation. Their transferability to Jiangyue donkey populations raised under different management conditions or with distinct genetic backgrounds therefore remains uncertain; extrapolation beyond the source population requires careful evaluation and external validation. Month-age was deliberately excluded as a continuous covariate to align the equations with their intended field application. Stratifying the study population into growing and adult groups reduced age-related variation, although the age range within each group may still have influenced the estimates. Precise birth records are often unavailable on farms; accordingly, the primary objective was to develop a body-weight estimation tool based exclusively on readily obtainable morphometric measurements. Despite the broad age range of the growing group (7 months to 3 years), the stepwise regression model retained age-associated traits such as chest circumference and body length and explained 91.8% of the variation in body weight (R^2^ = 0.918). Thus, much of the age-related variation may have been captured indirectly through these measurements, although an independent contribution of age cannot be excluded. Future studies should incorporate age as a covariate and use independent populations to assess whether model calibration and predictive performance can be improved. Nevertheless, the present equations retain practical value as field-based estimation tools where reliable age records are unavailable.

### 4.4. Fitting of Age-Related Weight Patterns with Different Models

Nonlinear models have been widely used to characterize body-weight growth in cattle [27], sheep [28], horses [29], poultry [30,31,32], and other livestock species, yet their application to donkeys remains comparatively limited. The four models evaluated here were selected because they are among the most established nonlinear functions for describing livestock growth. Of these, the Brody model attained the highest goodness of fit (R^2^ = 0.99960), closely capturing the population-level relationship between age and body weight in female Jiangyue donkeys aged 1–14 months. This result accords with that of Zhang et al. [33], who identified the Brody model as the best-performing model for Dezhou donkeys from birth to 18 months of age. By contrast, Li Xuanyue et al. [34] compared the Logistic and Gompertz models in Jiangyue donkeys aged 0–24 months and found that the Gompertz model provided the better fit. A similar ranking emerged here, with the Gompertz model outperforming the Logistic model. After the Brody and von Bertalanffy functions were included alongside the Logistic and Gompertz models, the Brody model provided the highest R^2^ and lowest RMSE, suggesting that it captured the gradual, moderate increase in body weight during early development more closely than the alternatives. These comparisons underscore the need to evaluate multiple candidate functions when selecting a growth model. Although the von Bertalanffy model yielded slightly lower AIC and BIC values, RMSE directly quantifies absolute prediction error and is therefore particularly relevant to weight estimation. On balance, considering both statistical performance and practical utility, the Brody model was selected as the preferred model. This interpretation nevertheless requires caution. The analysis was based on cross-sectional observations rather than repeated longitudinal measurements of the same cohort; accordingly, the fitted curves represent population-level age–weight relationships, not individual growth trajectories. Extrapolation beyond 14 months should therefore be avoided, particularly because adult animals were excluded from model fitting and the mean body weight of adult females in the present dataset was 228.02 kg.

## 5. Conclusions

Analysis of body weight and morphometric data from 484 female Jiangyue donkeys revealed highly significant positive correlations among all measured body dimensions. The particularly strong association between withers height and rump height indicated closely coordinated vertical skeletal growth and a high degree of proportionality in body conformation. Stepwise regression yielded equations for estimating body weight in growing (R^2^ = 0.918) and adult donkeys (R^2^ = 0.844), offering an accessible means of weight estimation on farms without electronic weighing equipment. Comparison of R^2^, RMSE, AIC, and BIC identified the Brody model as the best-performing model for describing changes in body weight between 1 and 14 months of age (R^2^ = 0.99960; RMSE = 5.868 kg). The underlying data were cross-sectional, however, rather than longitudinal measurements obtained from the same cohort. Moreover, because adult animals were not included in the fitting dataset, the estimated asymptotic parameter should not be interpreted as mature body weight. All models were derived from a single population; their generalizability across populations and management systems therefore requires validation using independent external datasets. Collectively, these findings provide a quantitative basis for precision feeding, genetic resource conservation, and selective breeding in Jiangyue donkeys.

## Figures and Tables

**Table 1:** Measurement methods of body weight and body measurement indicators.

Indicator	Unit	Measurement Content	Measuring Tool
Body weight	kg	Fasting weight before morning feeding	Platform scale
Withers height	cm	Vertical distance from the highest point of the withers to the ground	Measuring stick
Body length	cm	Straight-line distance from the anterior edge of the shoulder joint to the ipsilateral hip end	Measuring stick
Chest circumference	cm	Circumference of the body trunk measured vertically downward from the scapula for one full circle	Soft tape measure
Cannon circumference	cm	Horizontal circumference at the thinnest part of the cannon bone in the upper 1/3 of the left fore cannon	Soft tape measure
Head length	cm	Straight-line distance from the foremost end of the head to the midpoint of the line connecting the two ear roots at the posterior edge of the head	Soft tape measure
Neck length	cm	Straight-line distance from the occipital ridge to the anterior edge of the scapula	Soft tape measure
Chest width	cm	Width of the shoulder ends, i.e., the distance between the lateral angles of the two shoulder-humeral joints	Measuring stick
Chest depth	cm	Straight-line distance from the highest point of the withers to the lower edge of the chest	Measuring stick
Rump height	cm	Vertical distance from the highest point of the rump to the ground	Measuring stick
Rump length	cm	Distance from the hip bone to the hip end	Soft tape measure
Rump width	cm	Distance between the left and right hip bones	Measuring stick

**Table 2:** Nonlinear models for cross-sectional age–weight relationships.

Growth Curve Model	Formula
Logistic	Y = A/(1 + Be^−kt^)
Gompertz	Y = Ae^(−Bexp(−kt))^
Von Bertalanffy	Y = A(1 − Be^−kt^)^3^
Brody	Y = A(1 − Be^−kt^)

Note: Y is the body weight at t months of age; parameter A is the mature (asymptotic) value of body weight; parameter B is the time coefficient when the maximum growth rate is reached; k is the growth rate coefficient approaching the asymptotic body weight; and e is the base of the natural logarithm, approximately equal to 2.71828. The coefficient of determination (R^2^) was used to evaluate the fitting effect of the curve models. A larger R^2^ value closer to 1 indicates a better fitting effect of the curve.

**Table 3:** Phenotypic statistics of body weight and body measurements of growing female Jiangyue donkeys.

Trait	Mean	SD	CV	Min	Max
BW (kg)	173.86	33.103	19.04%	89	273
WH (cm)	121.70	5.948	4.89%	105	139
BL (cm)	122.24	8.135	6.65%	103	146
CC (cm)	124.74	8.211	6.58%	100	147
CAC (cm)	14.14	0.936	6.62%	12	18
HL (cm)	49.20	3.007	6.11%	42	58
NL (cm)	48.76	3.936	8.07%	36	61
CW (cm)	28.62	1.979	6.91%	23	35
CD (cm)	52.91	3.250	6.14%	44	62
RH (cm)	125.15	6.002	4.80%	107	143
RL (cm)	37.65	2.689	7.14%	30	45
RW (cm)	36.89	3.401	9.22%	23	47

BW = body weight; WH = withers height; BL = body length; CC = chest circumference; CAC = cannon circumference; HL = head length; NL = neck length; CW = chest width; CD = chest depth; RH = rump height; RL = rump length; RW = rump width.

**Table 4:** Phenotypic statistics of body weight and body measurements of adult female Jiangyue donkeys.

Trait	Mean	SD	CV	Min	Max
BW (kg)	228.02	36.479	16.00%	145	321
WH (cm)	125.88	7.223	5.74%	109	146
BL (cm)	130.26	8.301	6.37%	100	164
CC (cm)	136.81	7.693	5.62%	117	155
CAC (cm)	14.41	1.121	7.78%	12	18
HL (cm)	52.38	3.079	5.88%	42	61
NL (cm)	53.56	4.761	8.89%	42	66
CW (cm)	29.81	2.731	9.16%	22	38
CD (cm)	57.20	4.210	7.36%	48	70
RH (cm)	129.13	7.320	5.67%	109	148
RL (cm)	39.73	2.666	6.71%	30	47
RW (cm)	39.70	2.767	6.97%	30	48

BW = body weight; WH = withers height; BL = body length; CC = chest circumference; CAC = cannon circumference; HL = head length; NL = neck length; CW = chest width; CD = chest depth; RH = rump height; RL = rump length; RW = rump width.

**Table 5:** Correlation coefficients between body weight and body measurement indicators of growing female Jiangyue donkeys.

Trait	BW	WH	BL	CC	CAC	HL	NL	CW	CD	RH	RL	RW
BW	1											
WH	0.762 **	1										
BL	0.776 **	0.707 **	1									
CC	0.929 **	0.786 **	0.730 **	1								
CAC	0.627 **	0.630 **	0.544 **	0.577 **	1							
HL	0.652 **	0.579 **	0.577 **	0.623 **	0.548 **	1						
NL	0.517 **	0.537 **	0.430 **	0.533 **	0.264 **	0.371 **	1					
CW	0.719 **	0.561 **	0.562 **	0.669 **	0.544 **	0.530 **	0.395 **	1				
CD	0.540 **	0.591 **	0.534 **	0.664 **	0.310 **	0.319 **	0.355 **	0.350 **	1			
RH	0.787 **	0.924 **	0.724 **	0.798 **	0.646 **	0.611 **	0.505 **	0.612 **	0.559 **	1		
RL	0.851 **	0.727 **	0.713 **	0.794 **	0.606 **	0.620 **	0.512 **	0.654 **	0.391 **	0.764 **	1	
RW	0.838 **	0.677 **	0.647 **	0.813 **	0.565 **	0.689 **	0.447 **	0.656 **	0.450 **	0.701 **	0.809 **	1

** indicates statistically significant correlation (*p* < 0.01).

**Table 6:** Correlation coefficients between body weight and body measurement indicators of adult female Jiangyue donkeys.

Trait	BW	WH	BL	CC	CAC	HL	NL	CW	CD	RH	RL	RW
BW	1											
WH	0.748 **	1										
BL	0.736 **	0.680 **	1									
CC	0.904 **	0.801 **	0.700 **	1								
CAC	0.625 **	0.658 **	0.585 **	0.658 **	1							
HL	0.443 **	0.517 **	0.477 **	0.446 **	0.386 **	1						
NL	0.502 **	0.650 **	0.464 **	0.561 **	0.352 **	0.240 **	1					
CW	0.589 **	0.481 **	0.502 **	0.593 **	0.355 **	0.368 **	0.438 **	1				
CD	0.554 **	0.559 **	0.364 **	0.568 **	0.327 **	0.264 **	0.566 **	0.284 **	1			
RH	0.767 **	0.950 **	0.697 **	0.790 **	0.653 **	0.558 **	0.621 **	0.492 **	0.542 **	1		
RL	0.695 **	0.715 **	0.667 **	0.717 **	0.519 **	0.428 **	0.472 **	0.604 **	0.278 **	0.735 **	1	
RW	0.732 **	0.640 **	0.632 **	0.728 **	0.486 **	0.355 **	0.424 **	0.612 **	0.433 **	0.655 **	0.684 **	1

** indicates statistically significant correlation (*p* < 0.01).

**Table 7:** Regression models of body weight on body measurement traits of female Jiangyue donkeys at different growth stages.

Model	Body Measurement Traits	R^2^	Adjusted R^2^	Standard Error of Estimation	Durbin-Watson
Growing donkey	BL, CC, CW, CD, RL, RW	0.918	0.917	9.557	1.878
Adult donkey	BL, CC, RW	0.844	0.841	14.532	1.619

**Table 8:** Regression coefficient analysis of regression equation models for female Jiangyue donkeys at different growth stages.

Model	Item	Unstandardized Coefficient		Standardized Coefficient	t	Sig.	VIF
		B	Std. Error				
Growing donkey	Constant	−312.925	11.145		−28.077	<0.001	
	BL	0.614	0.109	0.151	5.659	<0.001	2.519
	CC	2.501	0.165	0.620	15.125	<0.001	5.957
	CW	1.472	0.401	0.088	3.671	<0.001	2.035
	CD	−0.863	0.248	−0.085	−3.483	0.001	2.094
	RL	1.807	0.422	0.147	4.281	<0.001	4.160
	RW	0.953	0.321	0.098	2.966	0.003	3.860
Adult donkey	Constant	−385.023	19.559		−19.685	<0.001	
	BL	0.774	0.185	0.176	4.182	<0.001	2.090
	CC	3.320	0.226	0.700	14.700	<0.001	2.673
	RW	1.461	0.579	0.111	2.526	0.012	2.269

**Table 9:** ANOVA of stepwise regression between body weight and body measurements of female Jiangyue donkeys at different growth stages.

Model	Item	Sum of Squares	df	Mean Square	F	Sig.
Growing donkey	Regression	296,873.209	6	49,478.868	541.738	<0.001
	Residual	26,395.386	289	91.334		
	Total	323,268.595	295			
Adult donkey	Regression	209,991.258	3	69,997.086	331.459	<0.001
	Residual	38,856.884	184	211.179		
	Total	248,848.142	187			

**Table 10:** Validation results of the body weight regression models for Jiangyue donkeys.

Population	Sample Size (Training/Test)	R^2^ (Training)	R^2^ (Test)	RMSE (kg)	MAE (kg)	MAPE (%)
Growing donkeys	236/60	0.915	0.933	8.32	6.83	4.28
Adult donkeys	146/42	0.843	0.847	13.71	11.88	5.39

**Table 11:** Repeated 10-fold cross-validation results for the body weight regression models.

Population	CV R^2^ (Mean ± SD)	CV RMSE (Mean ± SD, kg)
Growing donkeys	0.9187 ± 0.0560	9.32 ± 2.87
Adult donkeys	0.8476 ± 0.0679	14.44 ± 2.79

**Table 12:** Body weight data of female Jiangyue donkeys at different months of age.

Month of Age	Sample Size	Data Type	Body Weight (kg)
1	17	Mean	51.79
		Standard deviation	10.74
2	42	Mean	65.07
		Standard deviation	11.67
3	33	Mean	85.45
		Standard deviation	11.58
4	28	Mean	116.27
		Standard deviation	9.82
7	10	Mean	129.70
		Standard deviation	16.20
8	22	Mean	134.23
		Standard deviation	16.52
9	29	Mean	144.21
		Standard deviation	16.16
10	29	Mean	151.74
		Standard deviation	13.27
12	14	Mean	164.36
		Standard deviation	20.17
14	17	Mean	173.26
		Standard deviation	21.39

Data for 5, 6, 11, and 13 months of age were not included in the statistical analysis due to insufficient valid sample size.

**Table 13:** Nonlinear model fitting parameters for the cross-sectional age–weight relationship of female Jiangyue donkeys.

Fitting Model	Fitting Parameter			R^2^	*p* Value	RMSE (kg)	AIC	BIC
	A	B	k					
Logistic	173.593	2.668	0.307	0.96418	<0.0001	7.462	59.566	62.122
Gompertz	178.432	1.472	0.230	0.97165	<0.0001	6.639	56.292	58.848
Brody	191.049	0.844	0.145	0.99960	<0.0001	5.868	55.960	58.517
Von Bertalanffy	181.309	0.405	0.202	0.97398	<0.0001	6.360	55.090	57.647

Note: A is the asymptotic body weight (kg); B is the time coefficient at which the maximum growth rate is reached; k is the growth rate coefficient approaching the asymptotic weight; and R^2^ is the coefficient of determination, where a value closer to 1 indicates a better fit.

## Data Availability

The datasets analyzed during the current study are available from the corresponding author upon reasonable request.

## References

[1] Seyiti S., Kelimu A. (2021). Donkey industry in China: Current aspects, suggestions and future challenges. J. Equine Vet. Sci..

[2] Han R.L., Zhang X.Z., Li M. (2022). Research progress on the current status of donkey breed resources and molecular genetic breeding in China. Chin. J. Anim. Sci..

[3] Li Z.H., Xue F.R., Cui C., Meng L.J., Sun Z. (2025). The predicament and breakthrough strategies of donkey industry in China. China Anim. Ind..

[4] Zhang Z.W., Huang B.J., Wang Y.H., Zhu M., Liu G., Wang C. (2023). A survey report on the donkey original breeding farms in China: Current aspects and future prospective. Front. Vet. Sci..

[5] National Bureau of Statistics of China (2024). Livestock Farming: Donkey Inventory (2005–2024). https://data.stats.gov.cn/dg/website/page.html#/pc/national/fsYearData?cid=69c574ab128a44e595cc0b24502b771b.6b8e8719075f43d4bf83b261208b37b5.c4d82af16c3d4f0cb4f09d4af7d5888e.ea7b82974c00430887ce611dd101a2d6.3c993de07d1949c8bcd7e62aa2836fd8&dic=775da7c68f044483b176e66fae732b45.

[6] Cai C.Y., Zhou B.W., Wang K.X., Xue M., Liu G.Q. (2026). Current status, protection and utilization of donkey germplasm resources. Chin. J. Anim. Sci..

[7] Zhang Z.W., Huang B.J., Wang Y.H., Zhan Y.D., Ma Q.S., Li Q., Wang C.F. (2023). Research progress on donkey reproductive physiology and reproductive technology. Chin. J. Anim. Sci..

[8] Lyu C.P., Xiao G.L., Amuti K., Yili H.M. (2007). Preliminary study on the improvement effect of Jiangyue donkey. Heilongjiang J. Anim. Reprod..

[9] Lu D.L., Zhou X.L., Li J.F., Xu M., Zhang M., Zhou Y. (2019). Cultivation and research progress of Xinjiang Jiangyue donkey. Xinjiang Xumuye.

[10] China Animal Agriculture Association (2021). Technical specification for donkey performance testing. J. Anim. Ind..

[11] Teleken J.T., Galvão A.C., Robazza W.S. (2017). Comparing non-linear mathematical models to describe growth of different animals. Acta Sci. Anim. Sci..

[12] Liang Y., Zhu B., Jin S., Bao J., Xu L., Chen Y., Gao X., Zhang L., Gao H., Li J. (2018). The growth curve fitting and the correlation analysis between body weight and body measurements in Chinese Simmental beef cattle population. Acta Vet. Zootech. Sin..

[13] Feng X., Jiang Q., Feng Y., Wang Y., Chen Y. (2022). Growth curve fitting and correlation analysis of body weight and body measurements in Angus cattle. Acta Agric. Zhejiangensis.

[14] Zhang X., Yu X., Ge J., Wei C., Zhang M., Wang D., You Z., Huang X., Ma G. (2018). Fitting of the early growth curve for growing of Simmental cow in Xinjiang Area. Chin. J. Anim. Sci..

[15] Sun Q.Y., Mou W.Y. (2025). Analysis of factors influencing grain yield based on linear regression model—Taking Shandong Province as an example. Adv. Appl. Math..

[16] Wang P.J., Jiao D.C., Gao J.H., He S.Y., Guli B.H., Jing W.H., Zhao Y.Q., Chen J.B. (2007). Determination of body size and meat performance of Guanxin hybrid donkey. J. Domest. Anim. Ecol..

[17] Yang Y., Ji C.L., Li F.F., Li Z., Sun Y.J. (2018). Regression analysis of body weight and body size in Dezhou donkey. Heilongjiang Anim. Sci. Vet. Med..

[18] Xiao G.L., Lyu C.P., Yilihamu, Ainiwaer S. (2007). Correlation between body weight and body size traits in adult female Xinjiang donkeys. Dangdai Xumuye.

[19] Jin H., Wang B., Liu J., Zhao S.P., Jia Y.T., Li Q., Li Q.G., Xu J., Gao L., Xi H.L. (2025). Correlation analysis and stepwise regression equation establishment between body weight and body size of adult female Xin’anjiang buffalo. Chin. J. Anim. Sci..

[20] Ou Y.H. (2024). Analysis of Growth Performance and Growth and Development Patterns of Different Strains of Dezhou Donkey. Master’s Thesis.

[21] Nininahazwe P.C., Sow A., Roamba R.C., Kalandi M., Ahmed H.D., Ouedraogo G.A., Sawadogo G.J. (2017). West African donkey’s liveweight estimation using body measurements. Vet. World.

[22] Quaresma M., Bacellar D., Leiva B., Silva S.R. (2019). Estimation of live weight by body measurements in the Miranda donkey breed. J. Equine Vet. Sci..

[23] Xiao H.X., Moraniyazi N., Sulaiman A., Zhang G.T., Wufuer P., Xie L.R., Keyum M., Ajid T. (2022). Correlation analysis between body size and body weight of Jiangyue donkey in different years. Mod. J. Anim. Husb. Vet. Med..

[24] Zhao D.X., Wang K., He X.M., Lu Y., Fang Y.X., Liang C.X., He Z.C., Liu P.D., Yue D., Deng W.D. (2023). Correlation analysis between body weight and body size in Wenshan highland cattle cows. Chin. Cattle Sci..

[25] John P.A., Iyiola-Tunji A.O. (2020). Measures of relationships and association among body weight and biometric traits of donkeys in Northwest Nigeria. Niger. J. Anim. Prod..

[26] Xiao H.X., Aji D.T., Shi G.Q., Yu S.J., Shi P.S., Re X.T. (2012). Correlation and regression analysis of body weight and body size traits in Jiangyue donkey. China Dairy. Cattle..

[27] Si L.W.M., Cireng L., Nima C., Zhaxi D., Gesang Z., Basang P., Gama Y., Kong X.Y. (2025). Analysis of growth and development patterns of Tibetan yellow cattle, Jersey cattle and their crosses on the plateau. Feed Res..

[28] Zhang Z.L., Lyu Z.M., Liu W.J., Liu L.J., Wang J.J., Liu C.B., Lv S.L., Ni J.H., Wan J.C. (2026). Growth curve fitting analysis of crossbred improved population of South African Mutton Merino and Chinese Merino sheep. Heilongjiang Anim. Sci. Vet. Med..

[29] Shen Z.H., Wang C.K., Zeng Y.Q., Ouwang W., Wang P.M., Meng J., Yao X.K. (2024). Regression analysis of body weight and body size and growth curve fitting in Yili horse mares. Chin. J. Anim. Sci..

[30] Li X.L., Liu J.N., Liu X.H., Li H.J., Wang Y.X., Ge C.R., Wang K. (2025). Growth curve fitting analysis of hybrids between Tengchong Snow chicken and white feather broiler. Feed Ind..

[31] Li F.G., Wang C.Q., Liu T.C., Liu L., Mukeremu M.M.T., Yang Z., Zhang W.C., Wang H.E. (2025). Analysis of early growth and development patterns, correlation between body weight and body size, and egg quality in Yutian Ma duck. Heilongjiang Anim. Sci. Vet. Med..

[32] Chen S., Yang H.M., Zhu P.J., Han H.M., Zhao R.H., Xu T.L., Jia D.H. (2025). Growth and development patterns and growth curve fitting analysis of body weight in Jiangnan white goose. Feed Rev..

[33] Zhang Z.W., Gao X., Faheem M., Wang Y., Wang T., Shi X., Huang B., Zhu M., Wang C. (2022). Comparative analysis of growth and development characteristics of two Dezhou donkey strains. Livest. Sci..

[34] Li X.Y., Xiao H.X., Li C., Li J.H., Zhang J.L., Zhang W.H., Li X.B. (2022). Analysis of changes in body weight and body size of male and female Jiangyue donkeys at different months of age. Chin. J. Anim. Sci..

